# Expanding the boundaries of previously obtained informed consent in research: Views from participants in the Personalised Risk‐based Mammascreening study

**DOI:** 10.1111/hex.13746

**Published:** 2023-04-04

**Authors:** Jennifer E. Lutomski, Linda Rainey, Milou de Jong, Peggy Manders, Mireille J. M. Broeders

**Affiliations:** ^1^ Radboud Biobank Radboud University Medical Center Nijmegen The Netherlands; ^2^ Department of Health Evidence, Radboud Institute for Health Sciences Radboud University Medical Center Nijmegen The Netherlands; ^3^ Department of Data Stewardship Radboud University Medical Center Nijmegen The Netherlands; ^4^ Dutch Expert Centre for Screening Nijmegen The Netherlands

**Keywords:** cancer screening, focus groups, informed consent, personal autonomy, qualitative research

## Abstract

**Introduction:**

Understanding participants' concerns and information needs regarding broadened consent is crucial to ensure transparency and participant autonomy. Our study qualitatively examined these issues in women participating in the Personalized RISk‐based MAmmascreening study (PRISMA). The original PRISMA informed consent was project‐specific (i.e., breast cancer research), limiting the scope of secondary research. We explored participants' needs for broadened consent to preserve informed decision‐making while maximising the potential re‐use of data.

**Methods:**

Focus groups (FGs) were performed following a semistructured discussion guide. Two independent researchers analysed the data thematically using an inductive approach.

**Findings:**

Twenty‐three asymptomatic women and 13 women diagnosed with breast cancer were randomly divided into six FGs. Four superordinate themes were identified: (1) Normalization, (2) Attitude towards the pharmaceutical industry, (3) Privacy and (4) Knowledge. Our participants viewed data sharing as an important conduit for advancing medical science. Perceived integrity was more often attributed to noncommercial than commercial parties, with a marked mistrust towards the pharmaceutical industry. Most requested information needs related to data protection. Participants' ideal consent process would confer a range of options; for example, they would be able to choose with whom data can be shared, whether data will be de‐identified or anonymous, the expiration date of their consent and how, if requested, general and personal study results would be disclosed.

**Conclusion:**

Our participants expressed clear information needs and a strong desire to be actively engaged in future data sharing decisions. Given that many researchers collaborate with commercial parties, building public confidence in these institutions would be beneficial. Illustrative examples addressing privacy concerns and clarifying difficult terms would aid consent decision‐making. Although our participants displayed great altruism in sharing their data and accepted that broad consent would ultimately facilitate future research, broad consent did not reflect their ideal situation. Dynamic consent may be an option but warrants further feasibility research.

**Patient and Public Contribution:**

Women were recruited from the general breast cancer screening population. Their perceptions and information needs, as reported in this study, will not only inform broadened consent for PRISMA but ideally guide other consent templates and decisions regarding consent processes.

## 1 INTRODUCTION

Informed consent refers to the autonomous permission granted for an activity with the underlying premise that the individual fully comprehends what that activity entails.^1^ Informed consent has historically referred to a specific research question. However, dynamic and rapidly advancing research techniques coupled with the global expansion of data repositories and biobanks have rendered it nearly impossible to anticipate the breadth of future research. To stimulate the reuse of existing data and biomaterial, some data repositories and biobanks have resorted to broad consent.^2^ This form of consent covers future research that has not yet been defined or possibly even conceived at the time of participant recruitment. Broad consent has been deemed ethically acceptableprovided that participants are adequately informed.^3^ Critics contend, however, that broad consent is too vague and therefore precludes informed decision‐making. They purport that it is not truly informed consent but rather a generic permission that prioritises research interests over participant autonomy.^4^ This position is further bolstered by evidence suggesting that participants' comprehension of broad consent is suboptimal.^5^ Yet, given that many participants are supportive of research initiatives,^5^, ^6^ stakeholders in the field counter that trust in the institution may compensate for this inherent vagueness.^7^, ^8^ These conflicting viewpoints have consequently ignited debate over thresholds for informed decision‐making.^1^, ^6^, ^9^

Disconcertingly, there is no globally accepted consensus on the definition of broad consent. Following guidance from the World Medical Association's 2016 ‘Declaration on Ethical Considerations regarding Health Databases and Biobanks’,^10^ several stakeholders have drafted templates for broad consent.^4^, ^9^ Furthermore, there have been massive legal efforts undertaken in this sphere. To harmonize EU data protection laws, the General Data Protection Regulation (GDPR) was drafted and implemented in all EU Member States on May 25, 2018.^11^ Although this wide‐encompassing legislation has had profound effects on the sharing and use of data in medical research, it did not fully resolve the issue regarding the interpretation of broad consent.^12^ Thus, EU member states have adopted varied safeguards for the use of data,^13^ butoftentimes in the absence of clear national guidelines, local researchers and their institutions are left to define policies and requirements.^14^

Undoubtedly research participants should serve an integral role in this discussion, underscoring the need for qualitative research to guide the review and adaptation of broad consent templates and policies. Their concerns and information needs are key elements in accepting broad consent and data sharing in research.^15^ Since information needs may differ across research fields, exploring these dynamics within different subgroups of participants is essential. Thus, given this backdrop, we sought feedback from women who participated in the Personalised RISk‐based MAmmascreening (PRISMA) study.

The primary aim of PRISMA was to investigate the potential value of risk‐tailored versus traditional breast cancer screening protocols in the Netherlands. Mammograms (∼67,000), questionnaire data (∼38,000 surveys), blood (∼10,100 samples) and/or salvia (∼600 samples) were collected from participants, resulting in a unique cohort and a large population‐based biobank. The PRISMA study is a rich source of health research data that can likely serve as a biomarker pipeline for more than breast cancer alone. However, the original informed consent obtained from participants was project‐specific (i.e., research into breast cancer), hindering its use within the wider research community. Reapproaching women for broadened consent for other research would expand and encourage using these data. Yet, to ensure transparency and participant autonomy, it is first critical to understand PRISMA participants' concerns and needs regarding broadened consent. Thus, the primary aim of our study was to qualitatively explore PRISMA participants' perceptions of data sharing and their information needs for broadened consent to optimize informed decision‐making and garner confidence in the PRISMA data sharing initiative.

## 2 METHODS

### 2.1 Design

Focus groups (FGs) were performed following a semistructured discussion guide to explore PRISMA participants' perceptions of expanding the boundaries of their previous consent. Per the Regional Ethics Committee CMO Arnhem‐Nijmegen Ethics Committee (the Netherlands), this study fell outside the remit of the Dutch Medical Research with Human Subjects Law (*Wet Medisch‐wetenschappelijk Onderzoek met mensen* [Reference number: 2020‐6652]). Written informed consent was acquired before participation.

### 2.2 Setting

PRISMA is an observational prospective cohort study which was carried out in the Netherlands between 2014 and 2019. Since participants were recruited from the Dutch breast cancer screening programme, in line with screening eligibility criteria, all participants were asymptomatic females aged between 50 and 75 years. Four out of five country's regional screening organisations facilitated the PRISMA study (i.e., the North, South, Eastand South‐West screening regions). In 2020, PRISMA participant data were linked to the Dutch Cancer Registry to identify incident breast cancer cases (Netherlands Comprehensive Cancer Organisation).

### 2.3 Original PRISMA consent protocol

Participants in the PRISMA study were asked to consent to use their data and biomaterial for research into early detection, prognosis and treatment of breast cancer. They were further asked to consent to use their data and any residual biomaterial for ancillary studies into breast cancer; this included the possibility of genetic research. Participants could consent to (1) store their unprocessed screening mammogram and/or (2) complete an online questionnaire on breast cancer risk factors and/or (3) venous blood collection to obtain a DNA sample, blood serum and blood plasma and/or (4) collection of a saliva sample to obtain DNA. Informed consent was obtained before participation in the PRISMA study. A signed paper consent form was submitted at the screening site. The online questionnaire also began with a short consent form.

### 2.4 PRISMA Biobank

Biomaterials are stored at the Radboud Biobank, a hospital‐integrated central biobanking facility located within the Radboud University Medical Center (Radboudumc), Nijmegen, the Netherlands.^16^ Information regarding request procedures and material transfer agreements can be found at www.radboudbiobank.nl. In line with the facility's local policy, the collaboration between commercial partners and Radboudumc researchers is permitted for biomarker and genetic medical research and innovation. The use of biomaterial to produce commercial products is strictly prohibited.

### 2.5 Participants

Women were randomly selected from the PRISMA database using a digital random number calculator, stratified across asymptomatic participants and incident breast cancer cases. This stratification aimed to explore whether breast cancer status affects women's opinions of data sharing. Inclusion criteria included:

1. Consented to be approached for follow‐up studies,
2. Valid email address and,
3. In possession of a computer, laptop, telephone or tablet with a functional camera and microphone.

Personalised study invitation letters with an information leaflet were sent via email. In October 2020, invitations were sent to 200 asymptomatic women (50 per screening region) and 50 breast cancer cases (divided per region as equally as possible, with consideration given to regional follow‐up time). Due to the initial low response (16/250 women; 6.4%), a second batch of invitations was sent to an additional 200 asymptomatic women and 50 cases in November 2020. All women received one general reminder one week after the initial email invitation. Interested women contacted our research team, after which an FG date was scheduled and an informed consent form was mailed to the potential participant. The FGs were carried out between November and December 2020.

### 2.6 Procedures

Due to the Covid‐19 pandemic and subsequent government regulations, the FGs were performed digitally. We used Zoom,^17^ which serves as a highly suitable platform for qualitative data collection when compared with other video and conference technologies.^18^ All participants were provided with a short manual via email and offered help from a researcher (L. R.) in setting up Zoom on their laptop, tablet or telephone the week preceding the FG. All FGs followed a semistructured discussion guide and were led by one moderator with extensive experience in qualitative interviewing (L. R.).

The discussion guide, available in Supporting Information: 1, provides potential prompts to ensure topics of interest were addressed in the FGs. In brief, this guide includes open‐ended questions about the perceived benefits of data sharing, concerns regarding data sharing, parties with whom data can be shared, potential consequences of data sharing, information needs and procedural preferences. The notion of broadened consent was presented in its most general form to better understand participants' receptiveness to using their data by academic research institutions, pharmaceutical/medical companies and nonmedical commercial parties (such as department stores). Whereas the discussion guide served as a reminder of topics to address for the interviewer, the aim was to have the conversation between participants flow as naturally as possible. To avoid premeditated responses, the discussion guide was not provided to the participants before the FG discussions.

FGs were recorded and transcribed verbatim. FGs were organised until data saturation was achieved, that is, until no new themes were raised. Participants also completed a short demographic questionnaire.

### 2.7 Data analysis

**Figure 1:**
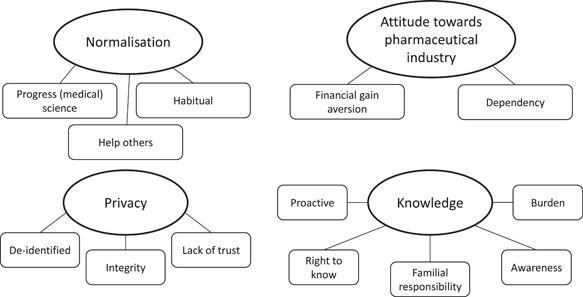
Final thematic map of perspectives on broadened consent: Themes and subthemes.

Data were thematically analyzed by two independent researchers (L. R., M. d. J.) using an inductive approach.^19^ Analysis followed six stages: familiarisation with the data, coding, developing themes, reviewing themes, defining and naming themes and final analysis.^19^ Consensus on the final thematic map (Figure 1) was reached through discussion. Descriptive analyses were performed with IBM SPSS Version 22 (IBM Corp.).

### 2.8 FAIR data statement

The authors are highly committed to FAIR data to ensure that data are Findable, Accessible, Interoperable and Reusable.^20^ Given our participants' concerns regarding maintaining their data, we have decided against placing the original transcripts in an open‐access repository. The original transcripts are available (in Dutch only) for reanalysis or meta‐synthesis purposes; email requests may be sent to: RadboudBiobank@radboudumc.nl

## 3 FINDINGS

Twenty‐three asymptomatic women (response: 5.8%) and 13 women who had been diagnosed with breast cancer (response: 13.0%) participated in our study. We organized six FGs ranging in size from four to seven women; on average, there were six women per group. The average age of participants was 62 years (SD: 7; range: 52–75 years). Most had a college/university degree (*n* = 24; 64.7%) and were married or lived with their partner (*n* = 27; 75.0%).

All participants were actively engaged in the FGs and many exhibited strong opinions on certain issues. Numerous topics from the discussion guide naturally emerged during the FG conversations without a prompt. In some instances, discussions of a specific topic reached a natural closure without organically progressing into a new topic. However, even when prompts were necessary, we found that the general flow of discussion was free and spontaneous with minimal silent pauses. The digital platform did not impede the FG discussionsand the average duration of the FGs was 58 min (range: 40–79 min).

The thematic map presented in Figure 1 provides an overview of PRISMA participants' perceptions of data sharing. Four superordinate themes were identified: (1) Normalisation, (2) Attitude towards the pharmaceutical industry, (3) Privacy and (4) Knowledge. The following FG excerpts illustrate the superordinate themes and corresponding subthemes (identified in italics). Supporting Information: 2 summarises all superordinate themes and their subthemes with relevant quotes.

### 3.1 Theme 1: Normalisation

Participants perceived data sharing as *part of the deal* when participating in scientific research. Their initial motivation to *progress (medical) science* had not wavered and they wanted their data to provide more relevant scientific insights. In the words of the participants, ‘More data mean more insights’, ‘More data mean more women will be helped in the future using more reliable statistics’, and ‘I think it's important that things are researched so solutions can be found and lives can be saved’,. They also felt a keen desire to *help others*, for example, ‘I am happy if I can help somebody’ and ‘But even if you don't have children, like me, I think it's important to pass information on to other girls, women, to assess whether you have cancer’. Women added that sharing personal information is ubiquitous in contemporary society, from supermarket discount cards to online cookies. Data sharing, therefore, feels *habitual* and relatively unremarkable, as illustrated by the following quotes, ‘We spend all day on the internet and I think Google knows more about us than we care to think about' and ‘I think sharing scientific data is safer than being on Whatsapp or Facebook’.

### 3.2 Theme 2: Attitude towards the pharmaceutical industry

Attitude towards the pharmaceutical industry was a major theme in the FGs and it was evident that topical events, namely the COVID‐19 pandemic, influenced this discussion. Participants expressed a great *aversion* towards sharing with pharmaceutical companies, particularly against their aim to make a profit, for example, ‘I don't want my data to be a source of income. It should lead to knowledge, not profit’, and ‘I'm a big believer in open source and with the current Covid‐19 pandemic you can see a lack of transparency from pharmaceutical companies whose only aim is to maximize profit. I wouldn't like it if my research data contributed to this’. Nonetheless, participants also recognized a certain *dependency* on pharmaceutical companies, with statements such as, ‘Considering the current Covid‐19 pandemic, there would be less hope for the future if we didn't have the pharmaceutical industry. So, it's an attachment and a dependency’ and ‘If pharmaceutical companies have more money than universities and can therefore develop better medical solutions, then yes…’.

### 3.3 Theme 3: Privacy

Concerns about privacy were raised frequently. In general, participants agreed that data sharing is acceptable as long as their data are de‐identified and the key to their identifiable information is kept at the university, for example, ‘If anonymity can be guaranteed, I'll allow data sharing’ and ‘I assume that the link to my name remains at the university medical centre’. Participants also emphasised the *integrity* of the research team, distinguishing between university researchers and commercial parties. As one participant stated, ‘I assume that researchers deal with your data in a decent and thorough fashion. I think that yes, researchers, yes are a different type of person to people who work for a commercial party’. They clearly voiced that their data should only be used for medical research, not commercial purposes, such as marketing. This was in part due to a *lack of trust* regarding privacy when sharing data with commercial and international parties, for example, ‘I don't want to be bombarded with commercial offers’, ‘What if you release your data commercially, where does it go?! I wouldn't know’, and ‘I believe that data security has been established in the Netherlands, however, I wouldn't want my data to be shared with the USA because that country leaks like a sieve’.

### 3.4 Theme 4: Knowledge

Choosing whether or not to be informed about future findings, particularly from genetic research, was critical for participants. Some felt that it is their *right to know* about personal genetic test results, as illustrated by, ‘Of course, you should be informed if you have a genetic anomaly; you allow them to test your DNA, then you should also be informed of the outcome’. Other participants emphasised the potential *burden* of such knowledge, for example, ‘I would rather not live with the thought that something bad is about to happen’. *Awareness* was mentioned as a reason to receive genetic test results, enabling an individual to put potential symptoms into perspective, for example, ‘You can recognise it and acknowledge it’. Women also felt a sense of *responsibility* towards other family members, as expressed in the following statements, ‘Some test results can be relevant for your brother, sister or children, passing this knowledge of would be an important first step. But that depends on the type of research’. Women also asserted that knowledge of genetic test results would allow them to act *proactively*, for example, ‘Yes, I would want to know. Not because I'd like to hear it, but because it would enable me to evaluate my life, act proactively and ultimately optimise my quality of life’.

### 3.5 Procedural preferences and information needs

Participants drew on both their professional and personal experiences when stating their procedural preferences and information needs. Although broadly posed, this question elicited quite specific responses. One participant who worked in the area of knowledge transfer, for example, mentioned the availability of a ‘socially responsible licensing toolkit’ to help translate academic knowledge into practice.

From a practical standpoint, our participants acknowledged the utility of an informed opt‐out consent procedure to enable optimal use of their data and biomaterial for medical research. This type of consent procedure implies that participants' data and biomaterial may be used for multiple studies in a similar research domain unless they explicitly object. It is often referred to as *informed* opt‐out since, in comparison to historical opt‐out procedures, greater measures are typically undertaken to communicate the scope of the consent to the participants. However, our participants emphasised the ability to partake in selective sharing would positively influence their willingness to participate in research studies. Participants would prefer to be able to choose:

1. The type of data that can be shared (i.e., mammogram, blood/saliva and/or questionnaire data in the case of the PRISMA study),
2. With whom the data can be shared (commercial vs. noncommercial),
3. Whether data are shared (inter)nationally,
4. The level of de‐identification/anonymity,
5. Whether to receive general study outcomes and/or personal (genetic) test results,
6. How feedback is reported and
7. How data sharing is handled after death.

They also stated a preference for illustrative examples to aid their understanding. PRISMA participants would like to update their consent after a previously agreed time period, for example after ten years, when new data are added to their source data or when conditions for data sharing or third parties change. Table 1 provides an overview of information needs that PRISMA participants would like to be included in the information letter accompanying the request to expand the boundaries of the previously obtained consent.

**Table 1:** PRISMA participants' information needs regarding expanding the boundaries of previously obtained informed consent.

Information needs
What conditions do a third party need to meet before my data will be shared?
With which parties will my data be shared?
What type of studies can be performed with my data?
What type of genetic research can be performed with my biomaterials?
Will my data be shared anonymously?
Why are commercial parties interested in my data?
Will I be informed of the general study results?
Will I be informed of personal test results, for example, genetic results? If not, please provide a reason.
Will my data be shared with integrity?
Will my data only be shared with parties whose primary goal is to progress medical science?
Will third parties be forbidden to pass my data on to other parties?
Who has the key to my identifying information?
Will all study results be published, including negative results?
Will the ‘Socially Responsible Licensing Toolkit’ be referenced?
How long will my data be stored?
How long will my consent be valid?
What happens with my data when I am deceased?

Abbreviation: PRISMA, Personalised RISk‐based MAmmascreening.

## 4 DISCUSSION

In our study, participants could reflect on their previous project‐specific consent when discussing under what conditions they would be willing to broaden their consent. Since this was not solely a hypothetical exercise, it was evident that their receptiveness to broadened consent was largely rooted in their underlying altruism for participating in the original PRISMA study, to improve screening practices and outcomes for women diagnosed with breast cancer. Our participants viewed sharing of their data and biomaterial as a conduit for advancing breast cancer research as well as medical research in general. Given the widespread use of digital monitoring technologies in their everyday lives, they experienced a normalization of data sharing, which they felt extended into scientific research. Yet, participants countered that data sharing should not come at the expense of privacy, as evidenced by most of their requested information needs relating to data protection. Perceived integrity was critical for our participants; integrity was more often attributed to noncommercial than commercial parties. They displayed a marked mistrust towards the pharmaceutical industry. Our participants emphasised that their ideal consent process would confer a range of options; for example, they would be able to choose with whom data can be shared, whether data will be de‐identified or anonymous, the expiration date of their consent and how, if requested, general and personal study results would be reported to them. Notably, participants diagnosed with breast cancer voiced similar opinions regarding data sharing to those who had not.

In general, our participants took a broad stance on re‐using their data and biomaterial. Whereas their original PRISMA consent referred to breast cancer research, our participants were not only open to the reuse of their data for other types of cancer research but also other (non‐cancer) medical‐related research, given that their procedural preferences and information needs were met. However, broad consent is not typically viewed through this lens. Ethicists and legal experts concur that broad consent does not imply complete unrestricted use of data and should, at minimum, be confined to research domains (for example, broadening breast cancer research to cancer research in general). The European Data Protection Board recently clarified this point in the context of the GDPR, stating that although Recital 33 GDPR may permit broad consent, the requirements stated in Article 5 GDPR (‘Principles relating to processing of personal data’) must still be observed.^12^ In brief, this means that even broad consent should have reasonable boundaries resembling the conditions under which the original data were collected.^12^ Furthermore, these boundaries should be in line with the expectations of the participants.^12^

In contrast to their openness regarding research outside the scope of breast cancer, our participants exhibited reservations regarding data sharing. Participants adamantly rejected using their data for nonmedical research; they felt that data sharing should only occur in the medical domain. They preferred medical commercial parties, namely pharmaceutical industries, to collaborate with academic institutions since they viewed the latter as more trustworthy. Thus, our participants did not entirely reject sharing their data and biomaterial with external parties but instead took a conditional stance (e.g., medical research only, cooperation with an academic partner).

Our participants' wariness towards medical commercial partners is not an isolated observation. Mistrust of commercial parties,^21^, ^22^ particularly amongst women^23^ is a widely recognised issue with potentially serious ramifications for medical research. In the United States alone, the industry supports more than half of all funded clinical trials and their sponsorship has led to numerous clinical advancements.^24^ Yet, restoring the general population's confidence in commercial parties requires a multifaceted approach. Numerous suggestions have previously been proposed,^24^, ^25^ and our research supports that serious consideration should be given to them. In brief, we should not expect participants to inherently trust commercial parties, but rather we should ensure the system prioritises safeguards against data misuse to gain participants' confidence.^25^

It was clear that the COVID‐19 pandemic, a highly publicised showcase of industry in action, patently influenced our FG discussions regarding data sharing with pharmaceutical companies. For some, the pandemic provided a concrete example of industry profiteering, thus underscoring the need for restricted data sharing. Whereas for others, it exemplified the pivotal role industry serves in global health, which is better achieved through data sharing. In overarching terms, the experience of the pandemic appeared to modestly improve the industry's favorability amongst our participants, yet not enough to abolish their pervasive underlying scepticism.

Optimal data security and privacy were marked concerns for our participants. Similar to their views on pharmaceutical research, our participants did not outrightly reject data sharing due to privacy concerns. They understood that data sharing could drive scientific research and innovation, but they placed a strong emphasis on the integrity of research teams and institutions. Data should only be shared with reputable parties prioritising privacy and data security and ideally linked with academic research. Although our participants displayed a high level of trust in their own national infrastructure for preserving privacy, there were clearly doubts regarding how international researchers would maintain their data. Mechanisms for data sharing and maintenance vary, with some more vulnerable to security risks than others. Cloud computing and virtualisation, however, are rapidly becoming the norm in the health sector.^26^, ^27^, ^28^ These advances have given rise to digital research environments, which permit secure, auditable data sharing and analysis within a virtual workspace,^29^ eliminating the need to transfer data between local hard drives. In the Netherlands, the AnDREa project is one of the numerous working examples of such endeavours,^29^ and PRISMA data will be accessible within this environment.

Nonetheless, it is questionable whether this form of data sharing is enough to assuage the fears expressed by our participants. Whereas cloud providers may be located locally, their data centres and server farms may be located worldwide. These information and commuications technology (ICT) concepts can be difficult to convey in information leaflets and potentially result in magnified fears over cyber security violations and confusion about where their data are maintained. Previous research in garnering confidence in digital data sharing in the healthcare sector can guide the development of suitable materials.^30^, ^31^

In regard to privacy, we observed an important conceptual issue relevant to participant communication. Participants often used the terms anonymous and de‐identified interchangeably. However, the difference between these terms is more than a semantic nuance; both options confer specific advantages and disadvantages. True anonymity would inhibit many of the participants' desired choices in the consent process. Participants should further be aware that not all data falls under privacy legislation. For example, Recital 26 GDPR stipulates that the legislation only covers de‐identified data (i.e., no identifiable information but can be traced back to the original participant via a code) not anonymous data (i.e., untraceable to the original participant).^32^ It is, therefore, crucial that these terms are clearly conveyed, ideally via illustrative examples as requested by our participants.

Our participants were interested in knowing both primary and unsolicited findings. Unsolicited findings, also referred to as incidental, co‐incidental or accidental findings, are unrelated to the primary research question but may be medically informative for a participant and/or family members. Reporting of unsolicited findings is carried out according to local policies and although broad guidelines have been designed, ultimately, local institutions may differ in opinion for medical actionability and may not always disclose results.^33^ The treatment of unsolicited findings remains a controversial topic; yet, our findings support the growing body of qualitative research that participants often wish to be informed.^34^, ^35^, ^36^ Local policies should acknowledge the participant's role in decision‐making. When prompted about genetic findings from their biomaterial, participants generally wanted to be active players in determining whether these findings were shared with them. Given that biobanking research does not always return results to participants, future researchers may wish to consider to what extent they can accommodate participants' preferences.

We observed numerous similarities between our participants' information needs and previous broad consent templates drafted without qualitative participant input.^4^, ^9^ These templates addressed noted concerns related to storage, privacy safeguardsand use/disposal of data after death. In the biobanking sphere, many local biobanks have expanded these templates and enacted strict policies regarding the use of data, imaging and biomaterial by commercial parties, another crucial issue raised by our participants. Still, our participants' demand for explicit information regarding future research studies more closely adheres to dynamic consent than broad consent. Dynamic consent capitalises on interactive interface technology to allow participants to consent to new research studies or update their consent status in real time; it can further be used as a portal for accessing personal study results.^37^ Dynamic consent is envisioned to increase participant engagement and autonomy,^38^, ^39^ and arguably, the reliance on broad consent may be viewed as archaic in light of such ICT advancements.^37^ Previous qualitative research exploring participants' views of a dynamic consent interface have been generally positive,^40^ which supports our interpretation that participants seek this type of consent process.

Yet, despite these findings, many challenges with dynamic consent persist. Consent fatigue is a noted concern that may lead to an increased number of study withdrawals.^1^, ^2^, ^39^ A potential option to promote autonomy (as desired by our participants) while reducing consent fatigue may be a hybrid opt‐in/opt‐out dynamic consent process. Opt‐in consent refers to participants actively consenting to using their data for research, whereas opt‐out consent refers to a process wherein participants must make an explicit objection. In this hybrid consent process, participants would complete a broad (opt‐in) consent form for future research. They would subsequently be notified via email each time their data and/or biomaterial are used, but they do not have to actively consent to future studies. Rather, they would have the option of opting out of specific studies via a link button in the email. Some data capture systems, such as Castor,^41^ have already developed dynamic consent modules which may be able to support this process, particularly if the data capture system is linked with the local biobank information management system. To the authors' knowledge, a hybrid consent procedure has not yet been carried out in practice. The feasibility of such a consent process would need to be evaluated prior to its endorsement.

A hybrid consent process, however, would only address one challenge in dynamic consent. If not already integrated into the standard ICT process flow within an institution, implementing and maintaining a dynamic consent portal is a major undertaking that may not be achievable in all settings. Moreover, maintaining a biobanking facility is a challenging endeavour in its own right, as it must adapt to evolving legal‐ethical requirements, preservation techniques and quality controls. Many biobank facilities are comprised of a small core team with limited capacity to take on extra administrative duties. Even a relatively minor activity, such as ensuring up‐to‐date participant contact details for dynamic consent, would pose a substantial burden for many biobanks. Thus, such an endeavour must be weighed against the utility.

Although our study participants have expressed interest in dynamic consent, in practice, its uptake may be limited.^39^ For example, most participants in the Rare Undiagnosed Diseases study^42^ who were offered dynamic consent never updated their consent decisions.^39^ Similarly, in the Extended Cohort for E‐health, Environment and DNA study,^43^ no strong support was found for adding a dynamic consent interface to an existing longitudinal cohort study with healthy volunteers.^38^ Participants were highly satisfied with their broad consent arrangement and had a high level of trust in the research team and their ability to securely share data.^38^ Interestingly, despite their high levels of trust in the PRISMA team, our participants generally wished to be more involved in data sharing decisions. This may be an artefact of cultural differences or selection bias between the source populations. Our FG participants represented a highly select population who chose to participate in breast cancer screening, the original PRISMA study and this subsequent qualitative research. Speculatively, these women are highly engaged in their own health‐related decision‐making and feel strongly about the importance of consent. Given these aforementioned issues in implementing dynamic consent, it would be prudent to conduct further feasibility and implementation research into its sustainability and practicality.

There were two main limitations in our study. Our response rate was low; however, our final sample size is comparable with other qualitative researchand data saturation was achieved within the FGs. Second, although participants were recruited from several screening centres across the Netherlands, our results might not represent all women eligible for breast cancer screening. Reassuringly, however, the information needs requested by our participants bared strong semblance to needs reported from other qualitative studies, thus strengthening our observations.

## 5 CONCLUSION

Our participants expressed very clear information needs and a strong desire to be actively engaged in future data sharing decisions. Given that many researchers collaborate with commercial parties and/or pharmaceutical companies, building public confidence in these institutions may be of benefit. Illustrative examples addressing privacy concerns and clarifying difficult terms would facilitate consent decision‐making. Although our participants displayed great altruism in sharing their data and accepted that broad consent would ultimately facilitate future research, broad consent did not reflect their ideal situation. Dynamic consent may be an option but warrants further feasibility research.

## AUTHOR CONTRIBUTIONS

*Concept and design*: Jennifer E. Lutomski, Linda Rainey, Peggy Manders and Mireille J. M. Broeders. *Acquisition, analysis or interpretation of data*: Linda Rainey and Milou de Jong. *Drafting of the manuscript*: Jennifer E. Lutomski and Linda Rainey. *Critical revision for intellectual content*: Jennifer E. Lutomski, Linda Rainey, Milou de Jong, Peggy Manders and Mireille J. M. Broeders. All authors read and approved the final manuscript. The corresponding author attests that all listed authors meet authorship criteria and that no others meeting the criteria have been omitted. All authors had full access to the data in the study and can take responsibility for the integrity of the data and the accuracy of the data analysis.

## CONFLICT OF INTEREST STATEMENT

The authors declare no conflict of interest.

## Supporting information

Supplementary information.

Supplementary information.

## Data Availability

Supporting Information: 1 provides the full interview guide. Supporting Information: 2 summarises all superordinate themes and their subthemes with relevant quotes. Full participant transcripts in Dutch are available upon request. Data requests should be submitted to the Radboud Biobank at RadboudBiobank@radboudumc.nl.

## References

[1] Grady C . Enduring and emerging challenges of informed consent. N Engl J Med. 2015;372(9):855‐862. 10.1056/NEJMra1411250 25714163

[2] Steinsbekk KS , Kåre Myskja B , Solberg B . Broad consent versus dynamic consent in biobank research: is passive participation an ethical problem? Eur J Human Genet. 2013;21(9):897‐902. 10.1038/ejhg.2012.282 23299918 PMC3746258

[3] Grady C , Eckstein L , Berkman B , et al. Broad consent for research with biological samples: workshop conclusions. Am J Bioeth. 2015;15(9):34‐42. 10.1080/15265161.2015.1062162 PMC4791589 26305750

[4] Salvaterra E , Lecchi L , Giovanelli S , et al. Banking together—a unified model of informed consent for biobanking. EMBO Rep. 2008;9(4):307‐313. 10.1038/embor.2008.41 18379580 PMC2288758

[5] Barnes R , Votova K , Rahimzadeh V , et al. Biobanking for genomic and personalized health research: participant perceptions and preferences. Biopreserv Biobank. 2020;18(3):204‐212. 10.1089/bio.2019.0090 32302503

[6] D'Abramo F , Schildmann J , Vollmann J . Research participants' perceptions and views on consent for biobank research: a review of empirical data and ethical analysis. BMC Med Ethics. 2015;16:60. 10.1186/s12910-015-0053-5 26354520 PMC4563851

[7] Hawkins AK , O'Doherty K . Biobank governance: a lesson in trust. New Genet Soc. 2010;29(3):311‐327.

[8] Bosisio F , Barazzetti G , Koutaissoff D , Spencer B . Patients' decision to contribute to a biobank in the light of the patient‐recruiter relationship—a qualitative study of broad consent in a hospital setting. J Community Genet. 2021;12(1):15‐25. 10.1007/s12687-020-00479-z 32779150 PMC7846645

[9] Strech D , Bein S , Brumhard M , et al. A template for broad consent in biobank research. Results and explanation of an evidence and consensus‐based development process. Eur J Med Genet. 2016;59(6‐7):295‐309. 10.1016/j.ejmg.2016.04.002 27130428

[10] Word Medical Association . WMA Declaration of Taipei on ethical considerations regarding health databases and biobanks. 2016. Accessed June 27, 2022. https://www.wma.net/policies-post/wma-declaration-of-taipei-on-ethical-considerations-regarding-health-databases-and-biobanks/ 10.3917/jib.283.0113 29561093

[11] EUR‐Lex . Official Journal of the European Union L 119/1. Regulation (EU)2016/679 of the European Parliament and of the Council of 27 Aprilon the protection of natural persons with regard to the processing of personal data and on the free movement of such dataand repealing Directive 95/46/EC (General Data Protection Regulation). on the protection of natural persons with regard to the processing of personal data and on the free movement of such data, and repealing Directive 95/46/EC (General Data Protection Regulation). 2016. Accessed January 9, 2023. https://eur-lex.europa.eu/legal-content/EN/TXT/PDF/?uri=CELEX:32016R0679&from=EN

[12] European Data Protection Board . EDPB Documenton response to the request from the European Commission for clarifications on the consistent application of the GDPR, focusing on health research. 2021. Accessed January 9, 2023. https://edpb.europa.eu/sites/default/files/files/file1/edpb_replyec_questionnaireresearch_final.pdf

[13] Kindt E , Czarnocki J , Kanevskaia O , Herveg J , López CAF . Study on the appropriate safeguards under Article 89(1) GDPR for the processing of personal data for scientific research: Final Report (EDPS/2019/02‐08). 2021. Accessed January 9, 2022. https://edpb.europa.eu/system/files/2022-01/legalstudy_on_the_appropriate_safeguards_89.1.pdf

[14] Smilan LE . Broad consent—are we asking enough? Ethics Hum Res. 2022;44(5):22‐31. 10.1002/eahr.500140 36047277

[15] Sanderson SC , Brothers KB , Mercaldo ND , et al. Public attitudes toward consent and data sharing in biobank research: a large multi‐site experimental survey in the US. Am J Hum Genet. 2017;100(3):414‐427. 10.1016/j.ajhg.2017.01.021 28190457 PMC5339111

[16] Manders P , Lutomski JE , Smit C , Swinkels DW , Zielhuis GA . The Radboud Biobank: a central facility for disease‐based biobanks to optimise use and distribution of biomaterial for scientific research in the Radboud University Medical Center, Nijmegen. Open J Bioresour. 2018;5:2. 10.5334/ojb.36

[17] Zoom Video Communications Inc . Security guide. Accessed June 27, 2022. https://d24cgw3uvb9a9h.cloudfront.net/static/81625/doc/Zoom-Security-White-Paper.pdf

[18] Archibald MM , Ambagtsheer RC , Casey MG , Lawless M . Using zoom videoconferencing for qualitative data collection: perceptions and experiences of researchers and participants. Int J Qualitative Methods. 2019;18:160940691987459.

[19] Braun V , Clarke V . Using thematic analysis in psychology. Qual Res Psychol. 2006;3(2):77‐101.

[20] Wilkinson MD , Dumontier M , Aalbersberg IJ , et al. The FAIR guiding principles for scientific data management and stewardship. Sci Data. 2016;3:160018. 10.1038/sdata.2016.18 26978244 PMC4792175

[21] Middleton A , Milne R , Almarri MA , et al. Global public perceptions of genomic data sharing: what shapes the willingness to donate DNA and health data? Am J Hum Genet. 2020;107(4):743‐752. 10.1016/j.ajhg.2020.08.023 32946764 PMC7536612

[22] Ghafur S , Van Dael J , Leis M , Darzi A , Sheikh A . Public perceptions on data sharing: key insights from the UK and the USA. Lancet Digital Health. 2020;2(9):e444‐e446. 10.1016/S2589-7500(20)30161-8 32838250 PMC7380931

[23] Pahus L , Suehs CM , Halimi L , et al. Patient distrust in pharmaceutical companies: an explanation for women under‐representation in respiratory clinical trials? BMC Med Ethics. 2020;21(1):72. 10.1186/s12910-020-00509-y 32791969 PMC7424561

[24] Bauchner H , Fontanarosa PB . Restoring confidence in the pharmaceutical industry. JAMA. 2013;309(6):607‐609. 10.1001/jama.2013.58 23403686

[25] Graham M . Data for sale: trust, confidence and sharing health data with commercial companies. J Med Ethics. Published Online July 30, 2021. 10.1136/medethics-2021-107464 PMC10359563 34330796

[26] Ryu AJ , Magnuson DR , Kingsley TC . Why mayo clinic is embracing the cloud and what this means for clinicians and researchers. Mayo Clin Proc Innov Qual Outcomes. 2021;5(6):969‐973. 10.1016/j.mayocpiqo.2021.08.010 34632298 PMC8488458

[27] Stroud C , Gee AW , Bain L , eds. Neuroscience Data in the Cloud: Opportunities and Challenges: Proceedings of a Workshop. National Academies Press; 2020. 32049471

[28] Resende JS , Magalhães L , Brandão A , Martins R , Antunes L . Towards a modular on‐premise approach for data sharing. Sensors. 2021;21(17):5805. 10.3390/s21175805 34502696 PMC8433755

[29] AnDREa (Azure Digital Research Environment) . Accessed July 7, 2022. https://www.andrea-cloud.eu/

[30] Shen N , Strauss J , Silver M , Carter‐Langford A , Wiljer D . The ehealth trust model: a patient privacy research framework. Stud Health Technol Inform. 2019;257:382‐387. 30741227

[31] Peikari HR , T R , Shah MH , Lo MC . Patients' perception of the information security management in health centers: the role of organizational and human factors. BMC Med Inform Decis Mak. 2018;18(1):102. 10.1186/s12911-018-0681-z 30442138 PMC6238272

[32] GDPR.EU . Regulation (EU) 2016/679: recital 26 not applicable to anonymous data. Accessed July 11, 2022. https://gdpr.eu/Recital-26-Not-applicable-to-anonymous-data

[33] van der Schoot V , Haer‐Wigman L , Feenstra I , et al. Lessons learned from unsolicited findings in clinical exome sequencing of 16,482 individuals. Eur J Human Genet. 2022;30(2):170‐177. 10.1038/s41431-021-00964-0 34697415 PMC8821629

[34] Schoot V , Viellevoije SJ , Tammer F , et al. The impact of unsolicited findings in clinical exome sequencing, a qualitative interview study. Eur J Human Genet. 2021;29(6):930‐939. 10.1038/s41431-021-00834-9 33637888 PMC8187681

[35] de Boer AW , Drewes YM , de Mutsert R , et al. Incidental findings in research: a focus group study about the perspective of the research participant. J Magn Reson Imaging. 2018;47(1):230‐237. 10.1002/jmri.25739 28470774

[36] Christenhusz GM , Devriendt K , Dierickx K . Disclosing incidental findings in genetics contexts: a review of the empirical ethical research. Eur J Med Genet. 2013;56(10):529‐540. 10.1016/j.ejmg.2013.08.006 24036277

[37] Kaye J , Whitley EA , Lund D , Morrison M , Teare H , Melham K . Dynamic consent: a patient interface for twenty‐first century research networks. Eur J Human Genet. 2015;23(2):141‐146. 10.1038/ejhg.2014.71 24801761 PMC4130658

[38] Wallace SE , Miola J . Adding dynamic consent to a longitudinal cohort study: a qualitative study of EXCEED participant perspectives. BMC Med Ethics. 2021;22(1):12. 10.1186/s12910-021-00583-w 33563268 PMC7874652

[39] Teare HJA , Prictor M , Kaye J . Reflections on dynamic consent in biomedical research: the story so far. Eur J Human Genet. 2021;29(4):649‐656. 10.1038/s41431-020-00771-z 33249421 PMC7695991

[40] Teare HJ , Morrison M , Whitley EA , Kaye J . Towards ‘Engagement 2.0’: insights from a study of dynamic consent with biobank participants. Digit Health. 2015;1:2055207615605644. 10.1177/2055207615605644 29942545 PMC6001239

[41] Castor Electronic Data Capture . Accessed January 9, 2023. https://castoredc.com

[42] Javaid MK , Forestier‐Zhang L , Watts L , et al. The RUDY study platform—a novel approach to patient driven research in rare musculoskeletal diseases. Orphanet J Rare Dis. 2016;11:150. 27825362 10.1186/s13023-016-0528-6 PMC5101709

[43] John C , Reeve NF , Free RC , et al. Corrigendum to: cohort profile: extended cohort for E‐health, Environment and DNA (EXCEED). Int J Epidemiol. 2019;48(5):1734. 10.1093/ije/dyz175 31365084 PMC6857747

