# Supplementary material for: Expanding the boundaries of previously obtained informed consent in research: Views from participants in the Personalised Risk‐based Mammascreening study
Source: Health Expect. 2023 Apr 4;26(3):1308–17. doi: 10.1111/hex.13746 (PMC10154863; doi:10.1111/hex.13746)
Supplement: Supplementary file 1 — Supplementary information. [file HEX-26--s002.docx]

**Semi-structured Interview Guide**

*“Perceptions of PRISMA participants regarding data sharing and extending the boundaries of previously obtained informed consent”*

**General introduction**

*Between 2014-2019 we collected data on breast cancer risk factors (PRISMA study) You participated in this study. You may have completed an online questionnaire, provided a blood or saliva sample, or allowed us to store your unprocessed mammogram. With these data we will explore whether we can categorise women based on their breast cancer risk and provide them with a tailored screening programme based on this risk. This personalised programme could entail more intensive screening for women at increased risk, or less intensive screening for women with a lower risk.*

*A lot of women participated in the PRISMA study. In total, 43,000 women completed the questionnaire, 10,500 women donated blood or saliva and 35,000 women allowed us to store their mammogram. We really appreciate the time and effort it took to participate. Therefore, we want to optimise our use of these data. You have all consented to the use of your research data for (breast) cancer research. However, we could also share these data with other parties to enable more (scientific) research, not just into (breast) cancer, but also, e.g., cardiovascular disease or genetic research. Before we are allowed to do this, we have to ask your consent to extend the scope of the consent you previously provided. You can then indicate whether you would consent to data sharing or not.*

*We are very curious about any views you have on this and which potential advantages and disadvantages you see. That’s what we will be talking about today.*

**Potential topics:**

- Would you be willing to share your data?
- Which advantages do you perceive?
- Which disadvantages do you perceive?
- The PRISMA study collected questionnaire data, blood/saliva and mammogram data. These are three different data sources that we could share with other parties. Do you feel that sharing questionnaire data is the same as sharing biomaterials? Do they have the same perceived advantages/disadvantages?
- Have you thought about potential consequences of data sharing?
- If biomaterials like blood or saliva are shared for genetic research, a small or large genetic anomaly could be found. These anomalies illustrate a risk of disease. If such an anomaly is found in your DNA, would you want to know?
- There are different types of genetic anomalies. Some could result in recommendations regarding preventative measures or treatment; other anomalies result in an increased risk of disease, but nothing can be done to decrease this risk. Does the type of anomaly affect whether you would want to be informed?
- Sometimes an anomaly is found that we don’t understand very well yet. We don’t know what the consequences (if any) of this anomaly is for the person’s health. Would you want to be informed of such an anomaly?
- How would you like to be informed of a genetic test result (letter, phone, face-to-face)?
- What perceptions do you have when I say that we could share your research data with commercial parties?
- There are different types of commercial parties, for example well-known brands or shops like the HEMA, but also the pharmaceutical industry, and companies that produces mammograms for breast cancer screening. Do these examples elicit different thoughts/feelings or do they all feel the same?
- When are we allowed to share your data with other (commercial) parties? When are we not allowed?
- For what types of research may your data be used?
- What information do you need to be able to make an informed choice regarding the extension of your previously given consent?
