# Supplementary material for: Expanding the boundaries of previously obtained informed consent in research: Views from participants in the Personalised Risk‐based Mammascreening study
Source: Health Expect. 2023 Apr 4;26(3):1308–17. doi: 10.1111/hex.13746 (PMC10154863; doi:10.1111/hex.13746)
Supplement: Supplementary file 2 — Supplementary information. [file HEX-26--s001.docx]

| **Theme** | **Example of statements** |
| --- | --- |
| **Normalization** | |
| Progressing | “More data mean more insights.” |
| (medical) science | “More data mean more women will be helped in the future using more reliable statistics.” |
|  | “I think it’s important that things are researched so solutions can be found and lives can be saved.” |
|  | “It’s good when more things can be researched, because that leads to prevention and that’s important.” |
|  | “I think it’s useful, it can progress other studies.” |
| Helping others | “I am happy if I can help somebody. |
|  | “But even if you don’t have children, like me, I think it’s important to pass information on to other girls, women, to assess whether you have cancer.” |
|  | “I think it’s important for people I know, my children, others who may have to deal with disease, that research is possible, and that’s why I do it. I want to end disease, and if I can contribute to that, great!” |
|  | “I’m thinking of better mammography equipment. That would benefit all women. So yes, I would want to contribute to that.” |
|  | “I think it’s important for better data to be available for our offspring.” |
| Habitual | “We spend all day on the internet and I think Google knows more about us than we care to think about.” |
|  | “I think sharing scientific data is safer than being on Whatsapp or Facebook.” |
|  | “I don’t see the disadvantages, I’ve already consented to transfer of my personal data to the pharmacy and other medical specialists.” |
|  | “Yes, and our supermarket discount card…” |
|  | “All your personal information is already out on the street in this digital age”. |
| **Attitude towards pharmaceutical industry** | |
| Financial gain aversion | “I don’t want my data to be a source of income. It should lead to knowledge, not profit.” |
|  | “I’m a big believer in open source, and with the current Covid-19 pandemic you can see a lack of transparency from pharmaceutical companies whose only aim is to maximise profit. I wouldn’t like it if my research data contributed to this.” |
|  | “I think the words ‘commercial’ and ‘fair’ don’t go together.” |
|  | “Yes, thinking from the standpoint of the pharmaceutical industry, if there’s the aim to make a profit I don’t want my data to be shared. I don’t think that’s necessary.” |
|  | “Outrageous profits are ridiculous.” |
| Dependency | “Considering the current Covid-19 pandemic, there would be less hope for the future if we didn’t have the pharmaceutical industry. So it’s an attachment and a dependency.” |
|  | “If pharmaceutical companies have more money than universities and can therefore develop better medical solutions, then yes…” |
|  | “If we want medicines, then there will be financial gain. Production of medicines will never be done without making a profit.” |
|  | “If an industry uses data to better themselves, yeah that doesn’t sounds positive. But the question is: don’t their solutions also better society? That makes it difficult, striking a balance, I think.” |
|  | “It’s two-sided, it can be commercial, but it’s positive if it leads to new developments, because we can all, or I have profited from that in the past.” |
| **Privacy** | |
| De-identified | “As long as its anonymous, it’s fine.” |
|  | “If anonymity can be guaranteed, I’ll allow data sharing.” |
|  | “As long as my name’s not attached to the data, I don’t care.” |
|  | “I assume that the link to my name remains at the university medical centre.” |
|  | “As long as I know for sure that my name and address isn’t part of the data that’s shared.” |
| Integrity | “I assume that researchers deal with your data in a decent and thorough fashion. I think that yes, researchers, yes are a different type of person to people who work for a commercial party.” |
|  | “I assume my data will be anonymous; that’s a level of trust that you have when you participate.” |
|  | “You assume a certain level of integrity.” |
|  | “It’s also a matter of trust, isn’t it. I mean, you’re researchers and if no one participates there’s nothing to research, so there’ll be no progress. So if you want to share data, I assume this is done with care and integrity.” |
|  | “If it’s done with integrity…I have to trust the university.” |
| Lack of trust | “I don’t want to be bombarded with commercial offers.” |
|  | “I believe that data security has been established in the Netherlands, however I wouldn’t want my data to be shared with the US, because that country leaks like a sieve.” |
|  | “What if you release your data commercially, where does it go?! I wouldn’t know.” |
|  | “I find that a difficult issue, because the US deals with data differently. This make me question how well it is protected.” |
|  | “Yes, other international parties would need to have similar privacy regulations as the Netherlands, because there are some countries that do not meet those standards.” |
| **Knowledge** | |
| Proactive | “Yes, I would want to know. Not because I’d like to hear it, but because it would enable me to evaluate my life, act proactively, and ultimately optimise my quality of life.” |
|  | “Yes, you might be able to do something about it.” |
|  | “It enables you to take action if something’s the matter.” |
|  | “Would I want to know, yes, but only if I can do something about it.” |
|  | “Yes, I would want to know, because that knowledge can enable you to change your plans or do different things.” |
| Right to know | “Of course you should be informed if you have a genetic anomaly; you allow them to test your DNA, then you should also be informed of the outcome.” |
|  | “Yes, I assume I will be contact if I’m at increased risk for anything.” |
|  | “I would want to know about everything.” |
|  | “If you participate in scientific research, you assume that you’ll automatically be informed if something’s the matter with you.” |
|  | “If there’s nothing I can do, I’ll just carry on living, but I would want to know.” |
| Familial responsibility | “Some test results can be relevant for your brother, sister or children, passing this knowledge on would be an important first step. But that depends on the type of research.” |
|  | “It can be of value to someone in your family.” |
|  | “What kind of disease is it. Does it affect my children, for example?” |
|  | “I think it’s very important for my family to know all the details.” |
|  | “I would like to know if my children have an increased risk,” |
| Awareness | “You can recognise it and acknowledge it.” |
|  | “It might make you more aware.” |
|  | “I would like to know, it may allow you to put certain things in perspective and you may understand them better.” |
|  | “It can make you aware of certain symptoms you’re having. That can provide a sense of tranquillity.” |
|  | “You start to recognise things...” |
| Burden | “I would rather not live with the thought that something bad is about to happen.” |
|  | “You have to decide for yourself whether you want to be burdened with that knowledge.” |
|  | “No, I wouldn’t want to know. If I have no symptoms, then just let me live. Then I don’t have to worry about it either.” |
|  | “The anxiety and worry, no, I wouldn’t want to know.” |
|  | “I would have a problem with that, because at the moment you’re living unburdened.” |
